# Zebrafish C-reactive protein isoforms inhibit SVCV replication by blocking autophagy through interactions with cell membrane cholesterol

**DOI:** 10.1038/s41598-020-57501-0

**Published:** 2020-01-17

**Authors:** Melissa Bello-Perez, Patricia Pereiro, Julio Coll, Beatriz Novoa, Luis Perez, Alberto Falco

**Affiliations:** 1 Instituto de Investigación, Desarrollo e Innovación en Biotecnología Sanitaria de Elche (IDiBE), Miguel Hernández University (UMH), Elche, 03202 Spain; 2 Instituto de Investigaciones Marinas (IIM), Consejo Superior de Investigaciones Científicas (CSIC), Vigo, 36208 Spain; 3 Instituto Nacional de Investigaciones y Tecnologías Agrarias y Alimentarias (INIA), Dpto. Biotecnología, Madrid, 28040 Spain

**Keywords:** Antimicrobial responses, Virus-host interactions

## Abstract

In the present work, the mechanisms involved in the recently reported antiviral activity of zebrafish C-reactive protein-like protein (CRP1-7) against the spring viraemia of carp rhabdovirus (SVCV) in fish are explored. The results neither indicate blocking of the attachment or the binding step of the viral replication cycle nor suggest the direct inhibition of G protein fusion activity or the stimulation of the host’s interferon system. However, an antiviral state in the host is induced. Further results showed that the antiviral protection conferred by CRP1-7 was mainly due to the inhibition of autophagic processes. Thus, given the high affinity of CRPs for cholesterol and the recently described influence of the cholesterol balance in lipid rafts on autophagy, both methyl-β-cyclodextrin (a cholesterol-complexing agent) and 25-hydroxycholesterol (a cholesterol molecule with antiviral properties) were used to further describe CRP activity. All the tested compounds exerted antiviral activity by affecting autophagy in a similar manner. Further assays indicate that CRP reduces autophagy activity by initially disturbing the cholesterol ratios in the host cellular membranes, which in turn negatively affects the intracellular regulation of reactive oxygen species (ROS) and increases lysosomal pH as a consequence. Ultimately, here we propose that such pH changes exert an inhibitory direct effect on SVCV replication by disrupting the pH-dependent membrane-fusogenic ability of the viral glycoprotein G, which allows the release of the virus from endosomes into cytoplasm during its entry phase.

## Introduction

The fine-tuned response of the human plasma C-reactive protein (CRP) levels to infection, inflammation or trauma makes this predominant acute phase protein (APP) one of the most studied health biomarkers, and it has been associated with predictions for cardiovascular risk and disease^1–3^. In humans, CRP is the prototypic APP^2^. Thus, in response to an acute phase response (APR)-inducing stimulus, the pro-inflammatory mediator interleukin 6 (IL-6) mediates the production and release of CRP into the blood, primarily from the liver^4^. As a consequence, circulating CRP levels may increase by as much as 10^3^-fold from barely detectable basal concentrations^2^.

Human CRP is the canonical member of the pentraxin protein family^3,5,6^. Pentraxins are divided into two groups according to their primary sequence: short and long pentraxins. CRP, together with serum-amyloid P component (SAP), shape the former one^3^. Human CRP and SAP show high degrees of sequence identity (51%)^7^, analogous molecular structures and functions^6,8,9^ and overlapping ligand specificities^10,11^. Therefore, it is not surprising that short pentraxins show species-specific, strain-specific, gender-specific (i.e., hormonal-specific), and interchangeable acute phase reactivity^2,12–16^.

The polarized planar structure of the circulating CRP molecules with opposite ligand recognition and multifunctional effector faces defines the CRP as soluble pattern recognition receptors endowed with crucial innate immune activities^1,10^. It has been extensively reported that the human pentameric CRP can recognize and bind, in a Ca^2+^-dependent manner, the surface-exposed phospholipid heads, preferentially phosphorylcholine^17^. Phosphorylcholine works not only as a pathogen-associated molecular pattern (PAMP)^10,18–20^, but also as a danger-associated molecular pattern (DAMP)^18,21–24^. This phosphorylcholine-binding site of the soluble CRP is also involved in interactions with, for instance, oxidized low-density lipoprotein (LDL)^21^, nuclear materials (such as chromatin, histones, small nuclear ribonucleoproteins)^25,26^, and other compounds that may not contain phosphorylcholine but are abundant in bacteria^27^, fungi^28,29^ and parasites^30,31^.

In mammals, CRPs are usually triggered during both viral and bacterial infections^32^, although associated serum CRP level increases are more characteristic of bacterial infections, during which they increase by 3-fold logs, while viruses induce lower but significant 10^1^ serum CRP levels^2,32,33^. Furthermore, the few existing studies that have analysed C-reactive-like protein (CRP) levels in fish show moderate serum level increases in response to both bacterial and viral infections, suggesting an antiviral effect for CRPs^34–36^. For example, in common carp (*Cyprinus carpio*), the serum CRP levels increase up to 2-, 6- and 10-fold in response to *Aeromonas salmonicida*^37^, *Aeromonas hydrophila*^34^ and cyprinid herpesvirus-3 (CyHV-3)^35^ infections, respectively.

Further positive correlations between CRP levels and viral infections have been established in fish by transcriptional analysis. For instance, significant upregulation of *crp* gene expression in several immune- and non-immune-related tissues of diverse fish species has been revealed in response to viruses such as CyHV-3^35^, red seabream iridovirus (RSIV)^38–40^, viral haemorrhagic septicaemia virus (VHSV)^41,42^ and spring viraemia of carp virus (SVCV)^42,43^. Similarly, higher transcriptional expression of *crp* genes was observed in common carp treated with polyinosinic:polycytidylic acid (polyI:C, a compound that mimics viral dsRNA)^36^, in DNA-vaccinated rainbow trout (*Oncorhynchus mykiss*)^44^ and zebrafish (*Danio rerio*) embryos microinjected with an expression plasmid encoding the *il6* gene^42^, a cytokine that is upregulated in response to viral infections in humans^45^.

In this sense, our recent findings show that all previously identified zebrafish CRP1-7 isoforms^46^ confer isoform-dependent anti-SVCV protection *in vitro* and *in vivo*^47^ and exert unexpected anti-SVCV synergistic effects^47^ with 25-hydroxycholesterol (25-HOC)^48^. Recombinant CRP from tongue sole (*Cynoglossus semilaevis*) has also been reported to enhance host resistance to RSIV infection when intraperitoneally (i.p.) co-injected with the virus inoculum^40^. However, despite the great relevance for evolutionary immunology and therapeutic potential of CRPs, the underlying mechanisms for CRP antiviral effects are not yet known. The present work has been focused on these mechanistic aspects.

## Results

### CRP1-7 anti-SVCV activity targets host cells rather than the virus

**Figure 1:**
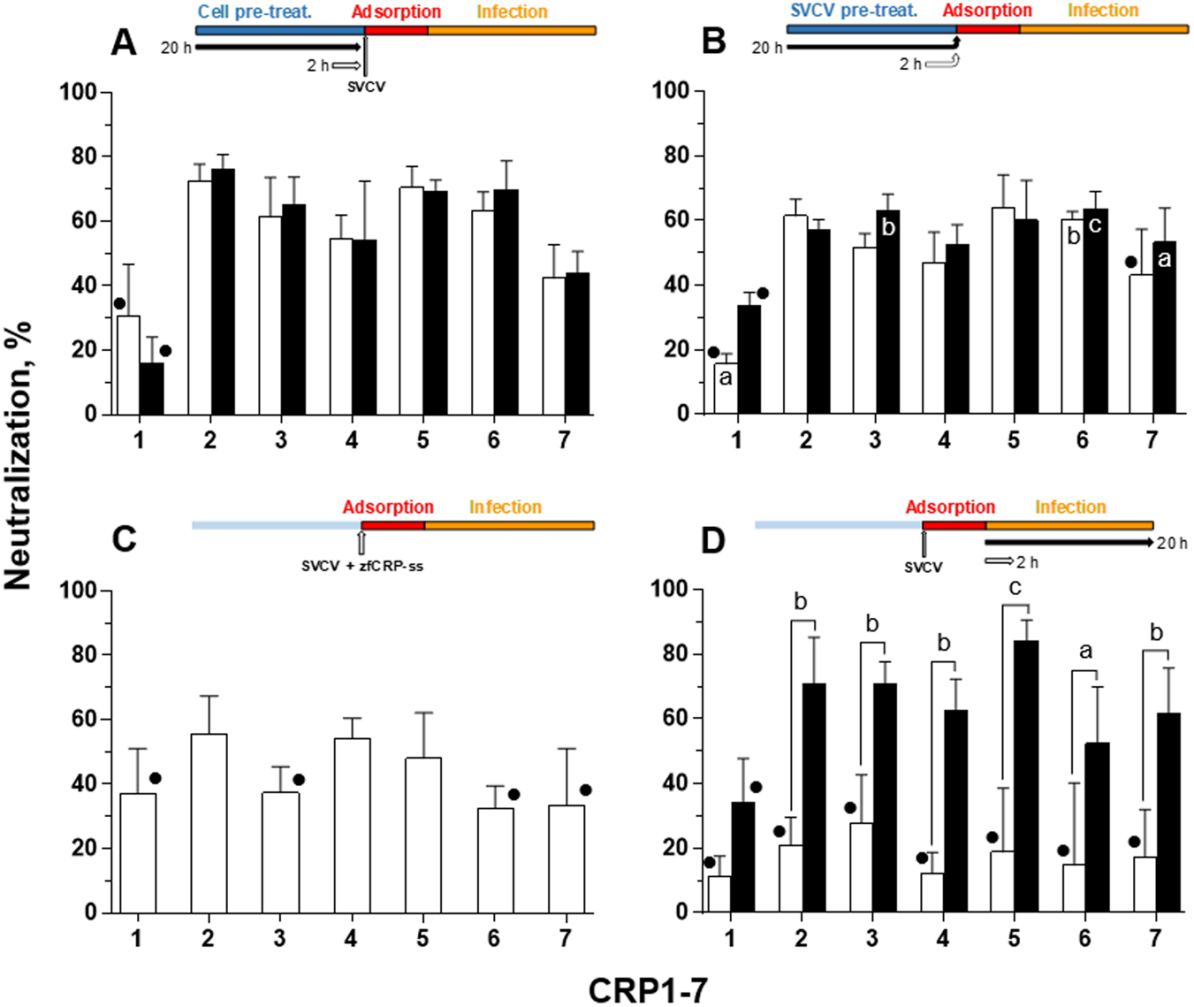
Effect of CRP treatment on SVCV replication in EPC cells. The neutralization activity of CRP1-7 was analysed by adding each CRP at different points of SVCV replication by incubating the CRP with (**A**) EPC cells before virus adsorption, (**B**) SVCV before and during virus adsorption, (**C**) both EPC cells and SVCV only during virus adsorption and (**D**) infected EPC cells (i.e., after adsorption). The duration of incubations was either 2 h (white bars) or 20 h (black bars). Descriptions of the experimental timeline charts are included as insets at the top of each corresponding graph. SVCV infection was determined by the focus forming assay. The data are expressed as percentages of neutralization. Graphs represent the mean and s.d. of three independent experiments, each performed in triplicate. • Indicates no significant differences between the treatment and the control (GFP treatment). The significant differences determined as *P* < 0.05, *P* < 0.01 and *P* < 0.001 were indicated as a, b or c, respectively. Inside-bar symbols from graph (**B**) indicate significant differences in comparison to corresponding CRP treatments in (**C**). Statistically significant differences between different times within the same CRP treatment are shown with symbols over the keys connecting both groups. Data were analysed by using two-way ANOVA with Sidak’s multiple comparisons test.

Our previous studies showed that the treatment with the supernatant from *epithelioma papulosum cyprinid* (EPC) cells that had been transfected with zebrafish CRP1-7 inhibited SVCV infection *in vitro*^42,47^; however, whether such anti-viral activity might be due to the interaction of CRP1-7 with viral particles remains to be demonstrated. To determine the stage in the viral cycle at which CRP1-7 might act, CRP1-7 treatments were added at different time points to SVCV-infected EPC cells (see diagram insets in Fig. 1 for further details). Thus, when either EPC cells (Fig. 1A) or SVCV (Fig. 1B) were treated with CRP1-7 prior to the viral adsorption stage, similar, significant inhibition of SVCV replication activities were observed for all CRPs (CRP2-6 inhibition was in the range of 47.1-76.2%), except for CRP1 and CRP7. In these assays, non-significant differences the different pre-treatment times (i.e., 2 and 20 h) were found, and pre-treatment at the 2 h time point already achieved high inhibition within these experimental settings (Fig. 1A,B). Additionally, moderate inhibition of SVCV replication was found when the treatments were restricted to the adsorption stage (Fig. 1C) as shown in Fig. 1B. Significant inhibitory effects were found for CRP2, 4 and 5 (55.6 ± 11.8%, 54.2 ± 6.2% and 46.6 ± 16.3%, respectively) in comparison to the control treatment (supernatant from EPC cells transfected with green fluorescent protein (GFP)) (Fig. 1C). Together, these results suggest that CRP antiviral activity may be due to a protective effect on EPC cells. In contrast, the duration of the treatment when added just after the adsorption stage significantly affected the inhibitory activity (*P* < 0.001); in particular, the inhibitory effect of CRP2-7 on SVCV replication was significantly increased when these treatments lasted 20 h (52.3–84.2%) in comparison to 2 h treatments (12.1–27.7%), in which the inhibition was not significantly greater than that of the corresponding GFP controls (Fig. 1D).

It should be noted that, using this same methodology, we also proceeded to determine whether the antiviral activity induced by CRP1–7 is actually due to the content of the CRPs in the treatment. For this purpose, the ligand binding capacity of each CRP for 25-HOC, which is described in our previous work^47^, was used to deplete each of the CRP isoforms in the CRP1–7 treatments. As observed in Supplementary Fig. S1, such depletion contributed significantly (*P* < 0.001) to decreasing the inhibitory infection capacity of the CRP treatments. The formulations individually tested with each CRP isoform showed that depletion of CRP2-6 significantly reduced the antiviral capacity compared to treatments without deletions; hence, since a direct correlation between anti-SVCV activity and CRP content could be established, the CRP2-6 were pooled (CRP-mix) for some of the experiments.

### Anti-SVCV protection conferred by CRPs is neither caused by hindered viral entry nor mediated by IFN

Although a time-dependent inhibitory activity observed in post-adsorption treatments (Fig. 1D) would indicate that late stages of the viral replication cycle were affected, this activity might also occur as a consequence of prolonged treatment that induces a continued protective state in the cells during viral infection and/or hinders virus entry at the appropriate steps during several consecutive replication cycles. Since the results obtained in the pre-treatment assays (Fig. 1A–C) already confirmed that any of these treatment effects are possible, subsequent efforts were focused on these possible mechanisms.

**Figure 2:**
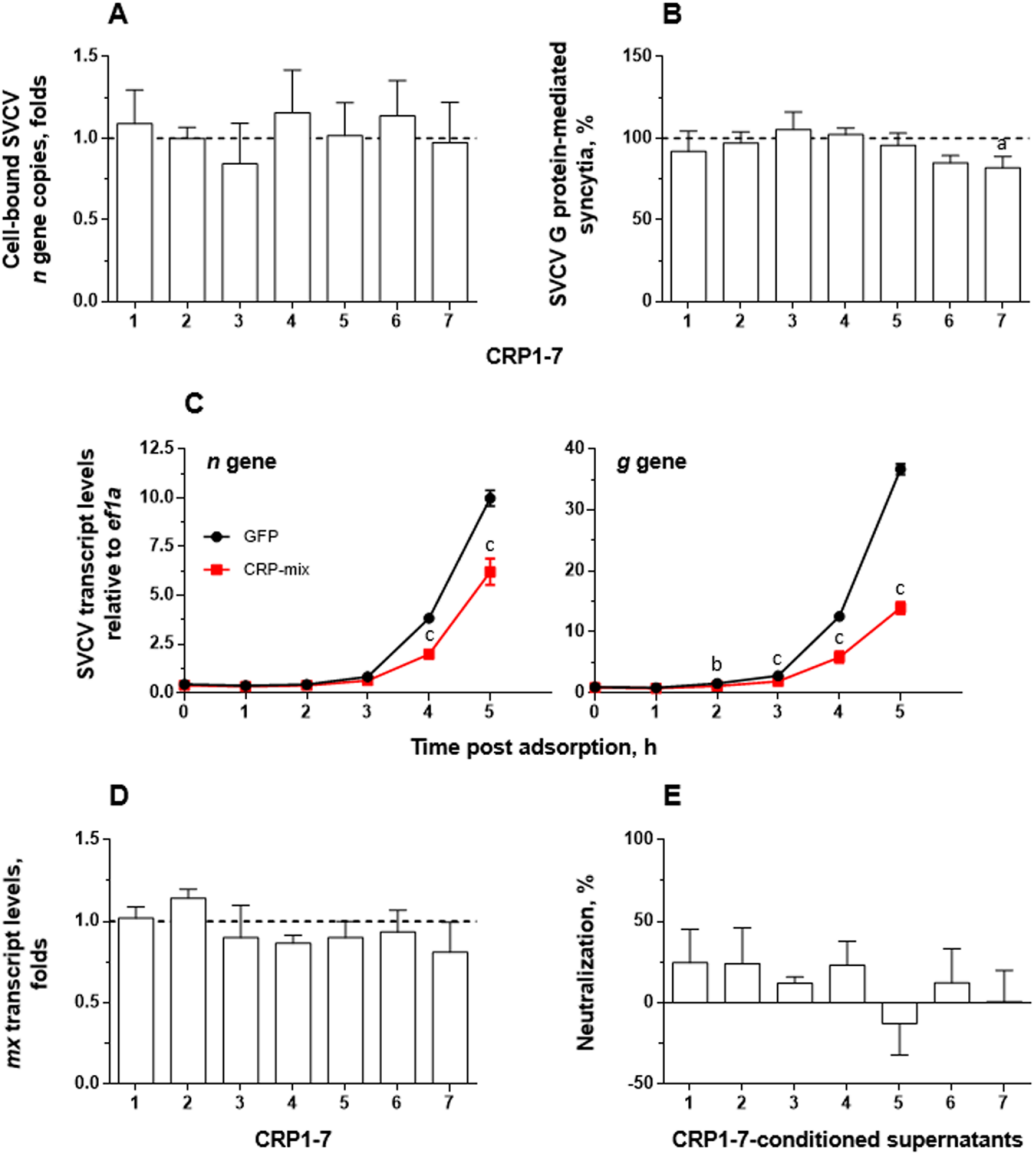
Interaction of CRP1-7 on SVCV replication in EPC cells. (**A**) SVCV binding levels to EPC cell surfaces in the presence of CRP1-7. EPC cell-bound SVCV particles in the presence of CRP were quantified by the number of SVCV *n* gene copies determined by RT-qPCR, and the data are expressed, relative to the number of *ef1a* transcripts, as fold changes. (**B**) CRP1-7 inhibition of the fusogenic activity of SVCV G protein on the surface of SVCV-infected EPC cells. The levels of G protein-mediated syncytia of 5 or more cells in SVCV-infected EPC cell monolayers were determined by triggering cell fusion at pH 6 in the presence of CRP and are expressed as percentage of the counted syncytia. (**C**) The time course of SVCV replication *in vitro* at early stages post adsorption. EPC cell monolayers were incubated for 2 h with the CRP-mix before viral adsorption, and the SVCV replication was estimated by measuring the expression of SVCV *n* and *g* gene transcripts by RT-qPCR and is expressed as fold changes. (**D**) Modulation of the IFN system by CRP1-7. The transcript levels of the IFN-response reporter *mx* gene were quantified by RT-qPCR in EPC cells 20 h after treatment with CRP for 2 h and were normalized to the corresponding *ef1a* levels. The data are expressed as fold changes. (**E**) Presence of antiviral factors in supernatants from CRP1-7-treated EPC cell monolayers. SVCV neutralization was induced by supernatants collected from EPC cells previously treated for 2 h with CRP1-7 and was determined by the focus forming assay. The results are expressed relative to GFP treatments. All experiments were performed 3 times each in triplicate, except for (**C**,**D**), which were performed twice each in quadruplicate. The data are presented as the mean and s.d. The significantly different levels between them are indicated with symbols as in Fig. 1. Data were analysed by using one-way ANOVA (**A**,**B**,**D**,**E**) and two-way ANOVA (**C**) with Sidak’s multiple comparisons test.

The initial steps of the rhabdovirus replication cycle comprise the attachment of the virions to the cell surface, binding of the rhabdovirus surface protein G to the host’s specific receptor/s, endocytosis and, finally, fusion of the viral and host endosomal membranes, which enables the release of the viral genome and associated proteins into the cytosol^49^. Therefore, to study the influence of CRP1-7 on the attachment/binding of the viral particles to the host cell membranes, the EPC cells were inoculated with SVCV at a multiplicity of infection (MOI) of 1, together with CRP1-7, and incubated for 2 h at 4 °C. After removing the non-attached viral particles, cell-bound SVCV was quantified by analysing the abundance of viral *n* gene copies as determined by reverse transcriptase quantitative polymerase chain reaction (RT-qPCR) (Fig. 2A). The results showed that the number of *n* gene copies remained invariable regardless of the CRP1-7 treatment used. The effect of each of the CRP1-7 on the pH-dependent fusion ability of SVCV protein G was studied by performing a fusion assay in which, by lowering the pH of the cell medium to 6, the fusion conformation of the SVCV G protein located at the membrane of previously infected cells triggered cell-to-cell fusion with the surrounding cellular membranes to generate quantifiable syncytia. The results showed that CRP1-7 did not exhibit any direct inhibitory effect on SVCV G protein-mediated membrane fusion, perhaps with the exception of CRP7 (which showed a fusion reduction of approximately 20% with *P* < 0.05) (Fig. 2B).

However, although the abovementioned assays demonstrated that CRP1-7 did not alter the virus entry step directly (Fig. 2A,B), the analysis of viral RNA synthesis at early post-adsorption stages (Fig. 2C), made by determining the levels of the viral *g* and *n* transcripts, showed that the treatment with CRP-mix decreased the expression levels of the viral genes as early as 4-5 h post adsorption, implying another inhibitory mechanism. For this reason, the ability of CRP1-7 to trigger the IFN system, the host’s typical and evolutionary-conserved response to viral infections^50^, was examined. However, the level of transcripts of *mx* remained at similar levels in all cases (Fig. 2D). Similarly, conditioned supernatants from EPC cells treated with CRP1-7 for 2 h and collected 20 h later, which would likely contain IFN if induced by CRP1-7, did not protect EPC cells from SVCV infection (Fig. 2E).

### CRP1-7 modulates the transcription of autophagy-related genes *in vitro* and *in vivo*

**Figure 3:**
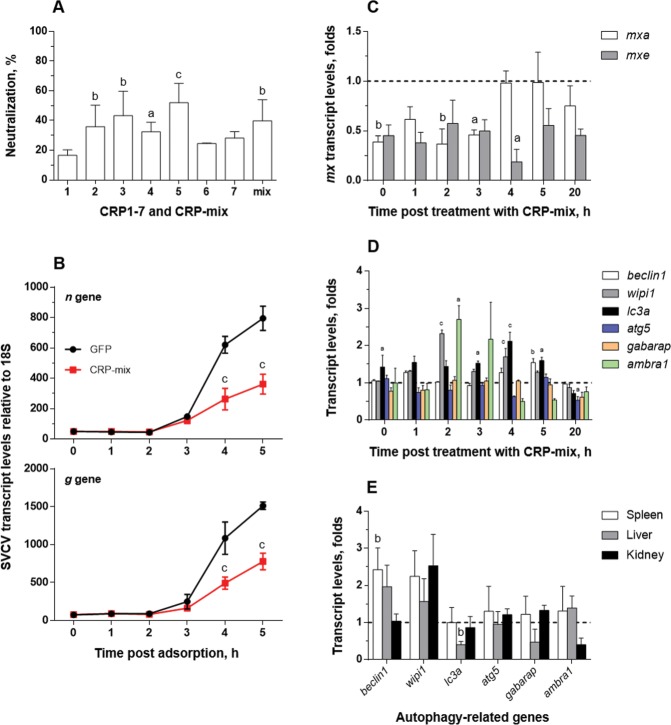
Interaction of CRP1-7 on SVCV replication in ZF4 zebrafish cells. (**A**) SVCV neutralization of CRP1-7 and CRP-mix when incubated with ZF4 cells for 2 h before virus adsorption. SVCV infection was determined by the focus forming assay. The results are represented as percentages of neutralization. These experiments were performed 3 times each in triplicate. (**B**) Time course of SVCV replication at early stages post adsorption. SVCV replication levels in ZF4 cells, incubated for 2 h with CRP-mix before viral adsorption, were determined at 0–5 h by measuring the expression of SVCV *n* and *g* gene transcripts by RT-qPCR. They are expressed as fold changes. (**C**) Induction of the IFN system by the CRP-mix. The transcript levels of the two IFN-response reporter gene isoforms of Mx (*mxa* and *mxe*) were quantified by RT-qPCR in ZF4 cells treated with the CRP-mix for 2 h before viral infection at different times post adsorption (0–5 and 20 h). The data were normalized to the corresponding 18S ribosomal levels and expressed as in Fig. 2D. (**D**) Capacity of the CRP-mix to modulate autophagy-related transcripts *in vitro*. The transcript levels of the relevant autophagy genes (*beclin1*, *wipi1*, *lc3a*, *atg5*, *gabarap* and *ambra1*) were quantified as described in in non-infected ZF4 cells (**C**). All gene expression studies were performed twice in quadruplicate *in vitro*. (**E**) Capacity of the CRP-mix to modulate autophagy-related gene transcripts *in vivo*. Four (non-infected) adult zebrafish were i.p. injected with the CRP-mix. Two days post injection, the transcript levels of the autophagy-related genes previously analysed *in vitro* were quantified by RT-qPCR in spleen, liver and kidney tissues. The data were normalized to the corresponding 18S ribosomal levels and expressed as fold changes. All data are presented as the mean and s.d. The statistically significant differences between them are indicated with symbols as indicated in Fig. 1. Data were analysed by using one-way ANOVA (**A**) and two-way ANOVA (**B**–**D**) with Sidak’s multiple comparisons test and multiple Student’s t-tests by the Holm-Sidak method (**E**).

Given that the mechanism by which CRP1-7 causes anti-SVCV neutralizing activity seems to promote an IFN-independent antiviral state, we proceeded to explore such observations in a homologous experimental system composed of the zebrafish-derived ZF4 cell line (also susceptible to SVCV infection), since the EPC cell line comes from fat-head minnow (*Pimephales promelas*), another fish species within the same family as the zebrafish (*Cyprinidae*)^51^. Thus, the CRP1-7 and CRP-mix pre-treatment of ZF4 cells for 2 h also conferred protection from SVCV infection (Fig. 3A), as in EPC cells (Fig. 1A). Likewise, the analysis of the progression of viral replication at early stages post adsorption in ZF4 *in vitro* (Fig. 3B) also exhibited an analogous profile to that observed in the EPC cells (Fig. 2C). For instance, the CRP-mix induced similar inhibition levels of SVCV replication in the ZF4 and EPC cells (≥2-fold at 4 h post adsorption).

In agreement with the data obtained using EPC cells, in ZF4 cells, the CRP-mix did not positively regulate the IFN response in SVCV-infected ZF4 (Fig. 3C). In contrast, the analysis of the transcriptional response of both *mxa* and *mxe* in zebrafish was significantly reduced over time by the CRP-mix (*P* < 0.001 for both *mxa* and *mxe*). Remarkably, the lowest levels of *mxa* (2.5-fold at 2 h post treatment) were restored to basal levels at 4 h post treatment, while the *mxe* levels did not fully stabilize after reaching their lowest levels (over 5-fold at 4 h post treatment), even at the latest post-treatment time point checked, which extended to 20 h in this set of experiments with the ZF4 cells. Additionally, the transcript levels of the genes encoding zebrafish IFNφ1 and 2 (*ifnphi1* and *ifnphi2*, respectively) showed similar profiles to the level of *mxa*, reaching upregulation levels at 5 (*ifnphi1*, 1.7 ± 0.04 folds, *P* < 0.01) and 20 h (*ifnphi1*, 1.8 ± 0.2, *P* < 0.01; *ifnphi2*, 2.1 ± 0.1, *P* < 0.01) from their corresponding lowest levels at 0 h (*ifnphi1*, 0.6 ± 0.1, *P* < 0.01; *ifnphi2*, 0.4 ± 0.04, *P* < 0.05) (Supplementary Fig. S2). However, such expression levels of IFN-response-related genes could not explain the observed antiviral protection rates^50^.

By analysing transcriptional expression, we proceeded to investigate whether autophagy, which had been relatively recently associated with an evolutionarily conserved antiviral protective response^52–54^, was involved in the neutralization of SVCV by CRP1-7. For this purpose, we initially chose to study the transcripts of some genes related to the autophagy pathway in non-infected ZF4 cells: *beclin1*, *wipi1*, *lc3a, atg5*, *gabarap* and *ambra1*. The results revealed that some of these relevant genes were stimulated by the CRP-mix in the ZF4 cells (Fig. 3D). In particular, the *wipi1*, *ambra1* and *lc3a* transcript levels were moderately elevated (1.5- to 3.5-fold; *P* < 0.05–0.001) during the initial stages after treatment with CRP-mix compared to control (GFP) treatments. These transcription levels started to stabilize 5 h post treatment and were fully restored after 20 h, except the level of *lc3a*, which was reduced (~2-fold, *P* < 0.05). Similarly, the analysis of the transcription levels of the abovementioned autophagy-related genes in immune-related tissues, such as spleen, liver and head kidney, after zebrafish were i.p. injected with the CRP-mix 2 days before the analysis, revealed that not only was autophagy transcriptionally modulated by CRPs *in vivo* but also this response was tissue-dependent. The highest levels were found for *beclin1* and *wipi1* in spleen and for *wipi1* in kidney (Fig. 3E).

### CRPs increase autophagosomes and modify their distribution in tissues

**Figure 4:**
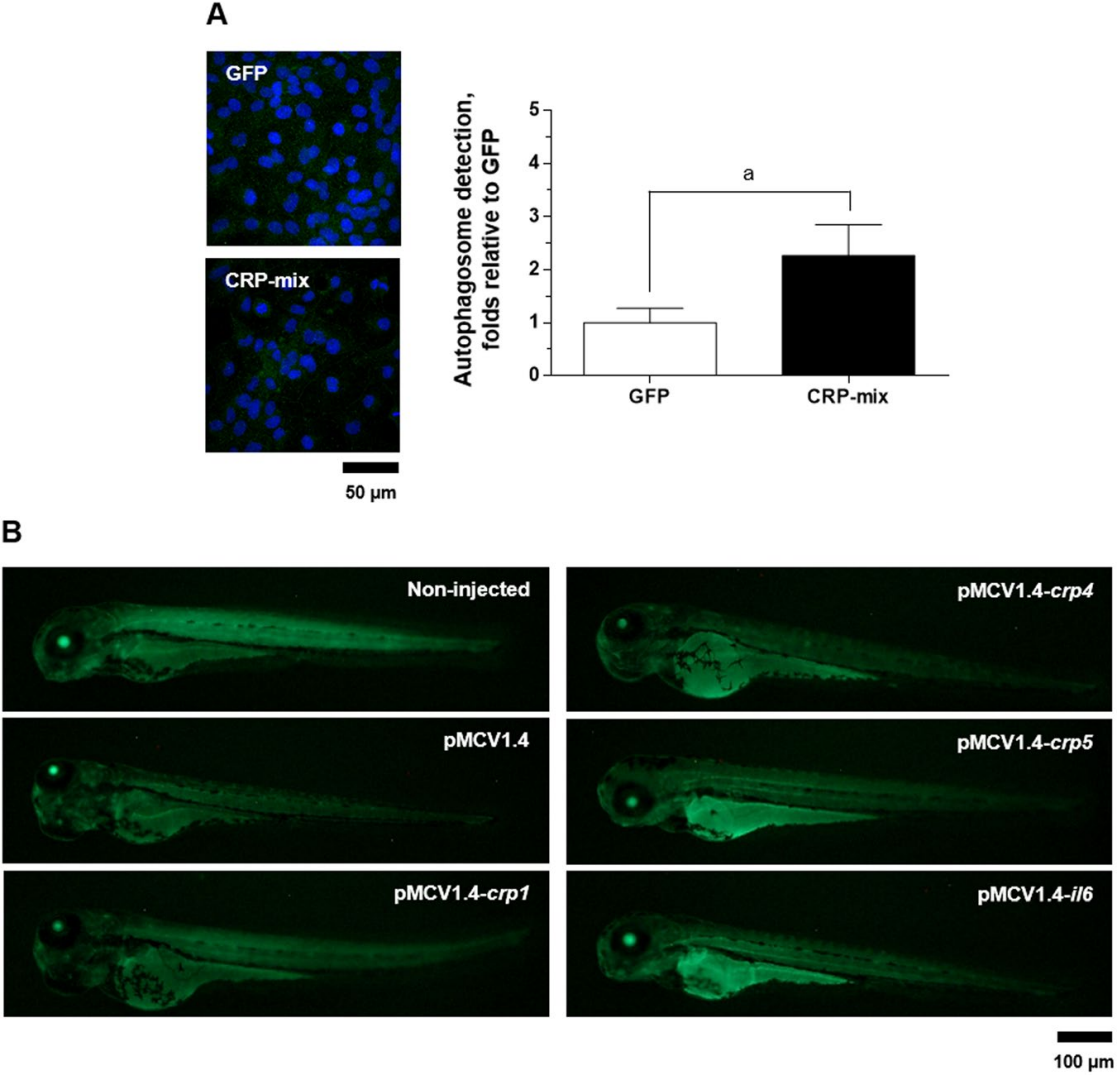
LC3 recruitment by selected CRPs in ZF4 cells and in zebrafish larvae. (**A**) Representative confocal images of the FITC immune-labelled LC3B in ZF4 cells treated with either GFP or the CRP-mix for 4 h. Nuclei were stained with DAPI. Autophagosome levels were quantified as the area (per cell) of over-threshold green fluorescence corresponding to the intracellular puncta and represented as fold changes in comparison to the GFP treatment as determined by the following formula: over-threshold fluorescence per cell in CRP-mix-treated monolayers/over-threshold fluorescence per cell in GFP-treated monolayers. This experiment was performed 3 times, each in triplicate. Symbol’a’ indicates statistically significant differences between CRP-mix and GFP treatments at the *P* < 0.05 level. Data were analysed by using two-tailed unpaired Student’s t-test. (**B**) Representative images of GFP-LC3 transgenic zebrafish larvae at 3 days post injection with 150 pg of pMCV1.4 or pMCV1.4-*crp1/crp4/crp5/il6* plasmid constructs. Corresponding scale bars equal 50 and 100 µm.

Autophagy levels were further studied by analysing the distribution of LC3 (a well-described autophagy marker)^55^ in ZF4 cells treated with CRP-mix and GFP. From the results obtained after microscopic quantification of the cytosolic LC3-positive fluorescent green-labelled puncta (a visible indicator of LC3 recruitment), autophagosome numbers had increased with CRP-mix treatments (2.3 ± 0.6-fold, *P* < 0.05) (see representative microscopic images and the resulting quantification graph in Fig. 4A).

To determine the influence of selected CRPs on the modulation of autophagy *in vivo*, the fluorescence of GFP-LC3 was visualized at low magnification in GFP-LC3 transgenic zebrafish larvae. For this experiment, one-cell embryo-stage zebrafish were microinjected 3 days before observation with the pMCV1.4 plasmid encoding zebrafish *crp1*, 4 and 5, as well as *il6*. The recombinant overexpression of CRPs resulted in increased fluorescence, especially in the yolk, indicating augmented basal autophagy levels in the yolk compared to larvae injected with empty plasmid (Fig. 4B). Among the *crp* genes tested, *crp5* was the most active in inducing such an effect; however, *il6* caused not only higher intensity but also more widely distributed fluorescence (Fig. 4B). Only LC3 fluorescence induced by *crp5* and *il6* was detected on the dorsal root ganglia. In this context, the analysis of *crp* expression after IL-6 induction revealed that, after the i.p. injection of IL-6 in EPC-transfected supernatants, the transcript levels of *crp3* (1.8 ± 0.1-fold, *P* < 0.001) and *crp5* (5.1 ± 0.8-fold, *P* < 0.01) significantly increased in zebrafish liver tissues, while the transcription levels of the other *crp* isoform genes remained unchanged (Supplementary Fig. S3).

### Inhibition of autophagy with CRPs inhibits SVCV infection

**Figure 5:**
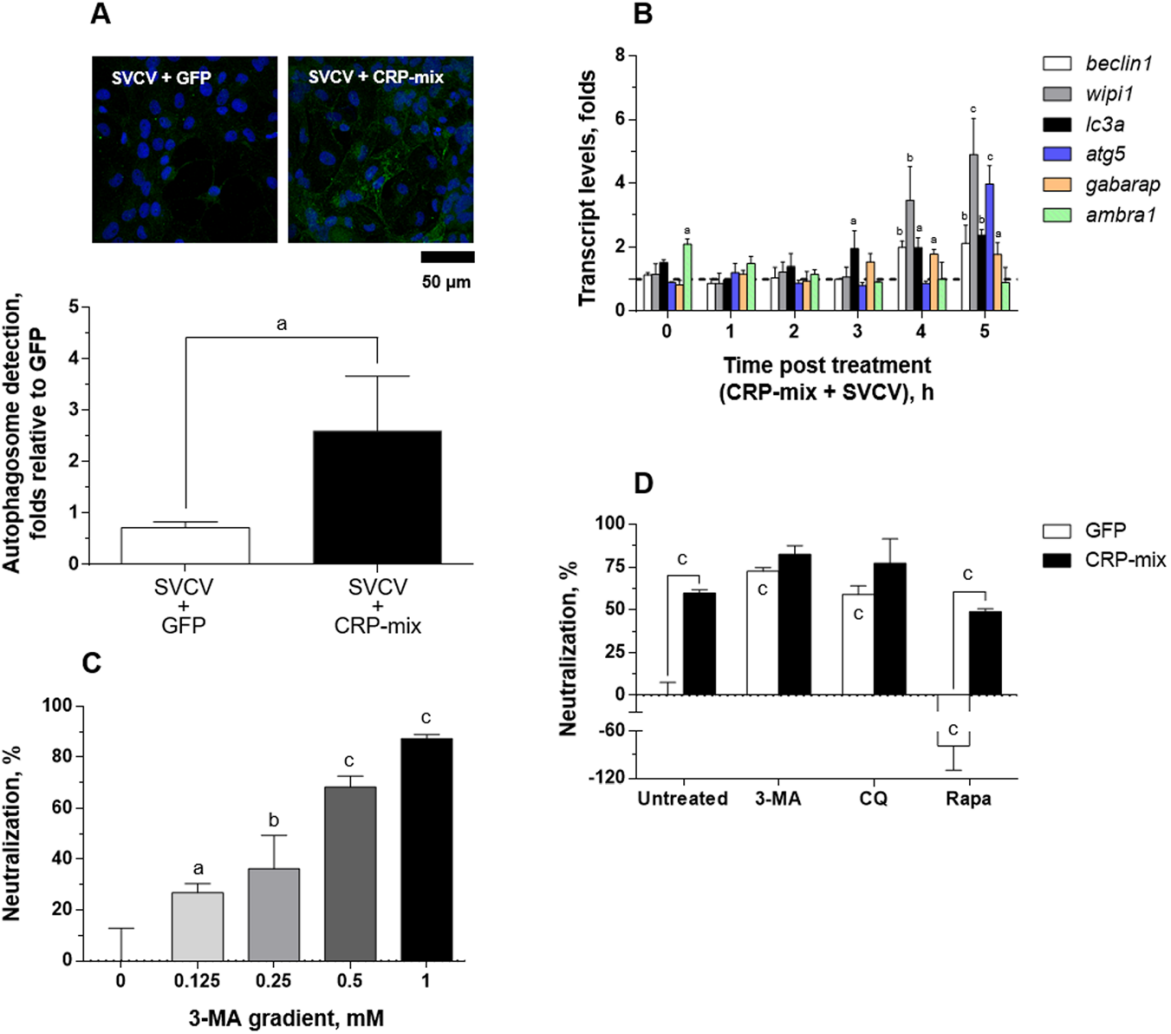
Autophagy induced by CRP-mix on SVCV replication in the ZF4 cells. (**A**) Representative confocal images of the FITC immune-labelled LC3B in the ZF4 cells treated with either GFP or CRP-mix together with SVCV for 4 h. Nuclei were stained with DAPI. Autophagosome levels were quantified as described in Fig. 4 and in the methods. The scale bar is equal to 50 µm. (**B**) Ability of the CRP-mix to modulate autophagy-related gene transcription *in vitro* during SVCV infection. The transcript levels of the genes of relevant autophagy elements (*beclin1*, *wipi1*, *lc3a*, *atg5*, *gabarap* and *ambra1*) were quantified by RT-qPCR in ZF4 cells treated with CRP-mix for 2 h before to viral inoculation (MOI of 1) at different times post adsorption (0–5 and 20 h). This experiment was performed twice in quadruplicate. The data are expressed as indicated in Fig. 3. (**C**) Effect of the autophagy blocker 3-MA on SVCV replication is shown. The SVCV neutralization activity of a gradient of 3-MA (0–1 mM) when incubated with EPC cells for 20 h prior to virus adsorption was assessed. SVCV infection was determined by the focus forming assay. The results are represented as the percentages of neutralization relative to the untreated group. (**D**) Effects of the CRP-mix on the SVCV neutralizing activity of autophagy modulators *in vitro*. SVCV infectivity was assessed on EPC cells treated with 3-MA (1 mM, 20 h), CQ (25 μM, 30 min) and rapamycin (Rapa, 25 μM, 4 h) and then incubated for 2 h with the CRP-mix before infection. SVCV infection was determined by the focus forming assay, and the data are presented as in (**C**) and relative to the GFP-treated group. Statistically significant differences in comparison to corresponding untreated groups and GFP are shown inside and on top of the bars, respectively. Neutralization experiments were performed 3 times each in triplicate. The statistically significant level differences are indicated with symbols as indicated in Fig. 1. Data were analysed by using one-way ANOVA (**C**) and two-way ANOVA (**B**,**D**) with Sidak’s multiple comparisons test and two-tailed unpaired Student’s t-test (**A**).

The LC3 recruitment was also analysed in response to SVCV in the presence/absence of the CRP-mix *in vitro* (Fig. 5A). Thus, after infecting ZF4 cells with SVCV (MOI of 1) for 4 h, no modulation of autophagosomes was apparent (0.7 ± 0.1-fold) in comparison to the uninfected (GFP) control cells (1.0 ± 0.3). Further results showed that when SVCV infection was carried out in combination with the CRP-mix, the number of autophagosomes significantly increased (2.6 ± 1.1-fold) but remained similar to the number obtained when the CRP-mix treatment had been used alone (2.3 ± 0.6, Fig. 4A).

In line with these findings, the analysis of the transcript expression of the autophagy-related genes at early stages (0–5 h) after SVCV infection in the presence of the CRP-mix (Fig. 5B) *in vitro* revealed that the presence of SVCV caused a 2 h delay in the transcriptional modulation observed with CRP-mix treatments (Fig. 3D). In contrast, the presence of SVCV did not reduce the transcription levels of any of the autophagy-related genes tested as the transcription levels were increased for *wipi1* (3.5 ± 1.1-fold at 4 h, *P* < 0.01; 4.9 ± 1.1-fold at 5 h, *P* < 0.001) and *atg5* (4.0 ± 0.6-fold at 5 h, *P* < 0.001). Regarding *lc3a*, significantly increased levels were already detected at 3 h (1.9 ± 0.6-fold, *P* < 0.05) and remained high until the end of the time course (2.0 ± 0.3-fold at 4 h, *P* < 0.05; 2.4 ± 0.2-fold at 5 h, *P* < 0.01). These results are also in contrast with the almost negligible transcript levels found for these genes in an identical time-course experiment but without CRP treatment (Supplementary Fig. S4). In the latter case, significant reduction in expressions was observed only for *wipi1* and *lc3a* at 0 h (~2-fold in both cases in comparison to non-infected cells).

Although the results described above suggest that CRPs might induce autophagy, this is a debated issue for rhabdoviruses^54,56–59^. In this context, Fig. 5C shows that the pre-treatment of ZF4 cells with 3-methyladenine (3-MA), an inhibitor of pI3K-III and therefore an autophagy inhibitor^60^, neutralized SVCV replication in a concentration-dependent manner, reaching neutralization levels of 87.4 ± 1.6% at the maximum concentration used (1 mM for 20 h), thus confirming the requirement for autophagic processes during SVCV replication. In turn, this result also suggests, at least in the present case, that the true effect of the CRPs on autophagy is inhibitory. To test this hypothesis, the ability to neutralize the infection with SVCV was used as a functional assay in combination with autophagy inhibitors 3-MA and chloroquine (CQ, inhibitor of lysosome/endosome fusion)^61^ and enhancer rapamycin (acting on mTOR)^55^. Thus, Fig. 5D shows that, although the treatment with the autophagy inhibitors neutralized the SVCV infection (as it had already been shown for 3-MA in Fig. 5C), its effect was greater when in combination with CRP-mix treatments. However, the treatment with 25 μM rapamycin for 4 h favoured the replication of SVCV (neutralization levels dropped to -78.9 ± 30.9%), and neutralization was restored by the addition of the CRP-mix (50.8 ± 1.4%) (Fig. 5C).

### Antiviral 25-HOC and methyl-β-cyclodextrin (MBCD) also interfere with autophagic processes

By using the experimental approach described above to study the involvement of autophagy in the antiviral effect of CRPs, we tested whether this mechanism was also associated with the antiviral activity of 25-HOC^48,62^, which had already been shown to act synergistically with CRPs^47^. Additionally, since the regulation of cholesterol had already been linked to the modulation of autophagy^63^, the effect of MBCD, a molecule with cholesterol-binding properties^64,65^, was also tested.

**Figure 6:**
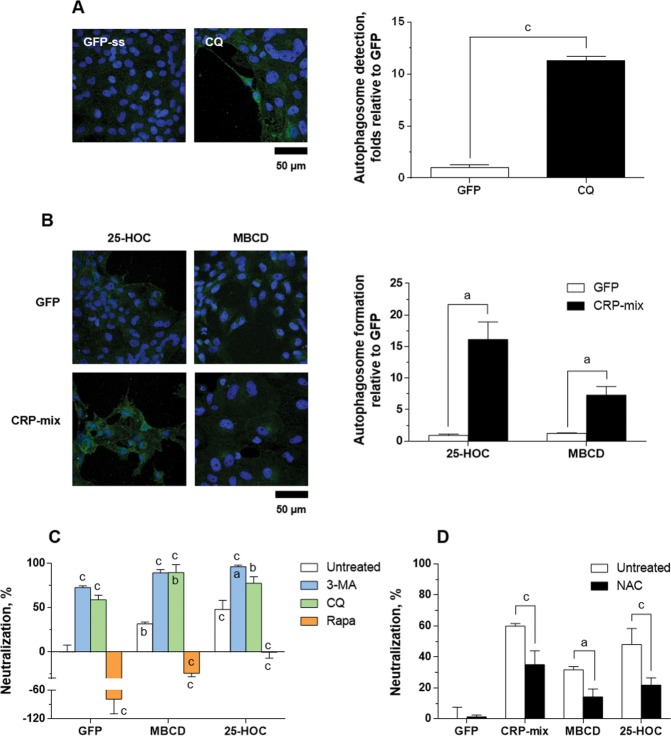
Autophagy and ROS generation during SVCV neutralizing activity induced by 25-HOC and MBCD together with the CRP-mix. Representative confocal images of the FITC immune-labelled LC3B in the ZF4 cells treated with (**A**) either GFP or CQ (25 µM) and (**B**) 10 μg/mL of 25-HOC or 4 mM MBCD alone or in combination with CRP-mix for 4 h. Nuclei were stained with DAPI. Autophagosome levels were quantified as described in Fig. 4 and in the methods. The scale bar is equal to 50 µm. (**C**) Effect of 25-HOC and MBCD on the SVCV neutralizing activity of autophagy modulators *in vitro*. SVCV infectivity was assessed for EPC cells treated with 3-MA (1 mM, 20 h), CQ (25 μM, 30 min) and rapamycin (Rapa, 25 μM, 4 h) and then incubated for 2 h with 10 μg/mL of 25-HOC or 1 mM MBCD before infection. SVCV infection was determined by the focus forming assay. Statistically significant differences in comparison to the corresponding GFP and untreated groups are shown inside and on top of the bars, respectively. (**D**) Effect of NAC on the SVCV neutralizing activity of the CRP-mix, 25-HOC and MBCD *in vitro*. SVCV infectivity was assessed for EPC cells treated with NAC (1 mM, 20 h) and then incubated for 2 h with either GFP, CRP-mix, 10 μg/mL of 25-HOC or 1 mM MBCD before infection. SVCV infection was determined by the focus forming assay. The results from the neutralization assays are represented as in Fig. 5. These experiments were performed 3 times in triplicate. All statistically significant level differences between treatment and corresponding control groups are indicated with symbols as in Fig. 1. Data were analysed by using two-tailed unpaired Student’s t-test (**A**,**B**) and two-way ANOVA with Sidak’s multiple comparisons test (**C**,**D**).

First, this methodology was validated by comparing GFP (1.0 ± 0.3-fold) to CQ treatments (11.3 ± 0.4-fold, *P* < 0.001) (Fig. 6A), an aforementioned autophagy inhibitor of the last steps of the autophagic process with an autophagosome cumulative effect^61^. In this regard, autophagosome levels for CQ solvent control, i.e., 2.5% ethanol, were 0.5 ± 0.1-fold (Supplementary Fig. S5A). Then, the ability to modulate the recruitment of LC3 was analysed in ZF4 cells in response to 25-HOC and MBCD in the presence/absence of CRP-mix. As Fig. 6B shows, after treating ZF4 cells with 25-HOC (10 μg/mL) or MBCD (4 mM) for 4 h, no modulation of the autophagosome was observed in any case (0.9 ± 0.2-fold for 25-HOC and 1.2 ± 0.2-fold for MBCD). In combination with the CRP-mix, upregulations were found for both compounds, 16.1 ± 2.8-fold for 25-HOC and 7.3 ± 1.4-fold for MBCD, in comparison to the corresponding treatments without the CRP-mix (*P* < 0.05).

SVCV neutralizing assays performed by combining either 25-HOC or MBCD with the autophagy modulators (Fig. 6C) showed that the combinations of both compounds with any of the autophagy inhibitors 3-MA or CQ increased their induced SVCV neutralization when added alone. In contrast, the autophagy-enhancer rapamycin, which increased SVCV infectivity when added alone (neutralization levels of -78.7 ± 30.9%), reverted the SVCV neutralization induced by both 25-HOC (from 48.2 ± 10.3% to -0.3 ± 6.9%, *P* < 0.001) and MBCD (from 31.7 ± 2.0% to -24.3 ± 3.9%, *P* < 0.001). Similarly, treatment with N-acetyl cysteine (NAC) (Fig. 6D), a hijacker of reactive oxygen species (ROS) with the ability to block/inhibit autophagy^66^, did not affect the replication of SVCV when used alone, but it did revert the inhibitory effect induced by the CRP-mix, 25-HOC and/or MBCD by ~50% (Fig. 6C).

### CRPs increase intracellular ROS and lysosomal pH levels

**Figure 7:**
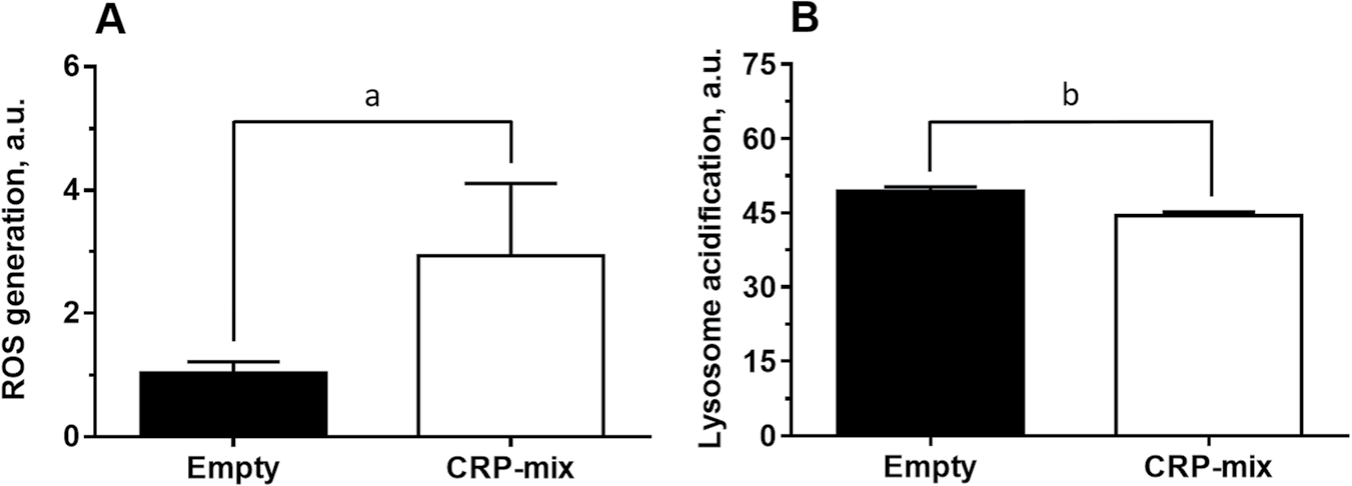
ROS generation and alkalization of intracellular pH induced by CRPs. (**A**) Effect of CRPs to generate oxidative stress *in vitro*. ROS formation was quantified in ZF4 cells transfected with pMCV1.4-*crp1-7* for 48 h and incubated for 30 min with the stress indicator CM-H2DCFDA. ROS generation was determined measuring fluorescence intensity (n = 4). (**B**) Ability of the CRP-mix to modulate the pH of lysosomes. Changes in the lysosomal pH were determined in ZF4 cells co-transfected with each *crp*-encoding plasmid for 48 h and stained with LysoTracker Green DND-26 for 30 min. The quantification of the green fluorescence was carried out by flow-cytometry (n = 6). Results are shown in arbitrary units (a.u.). All statistically significant level differences between treatment and corresponding control groups are indicated with symbols as in Fig. 1. Data were analysed by using two-tailed unpaired Student’s t-test.

The reversion of the anti-SVCV activity by CRPs as a consequence of the treatment with NAC suggests a role on the regulation of intracellular ROS levels. Indeed, it is reported that an increase in intracellular ROS levels exerts an alkalising effect on lysosomal pH and a subsequent inhibition of autophagy^67^. To check such hypothesis, we proceeded to analyse the ability of CRPs to modify both ROS and lysosomal pH levels in ZF4 cells. In this sense, considering the constitutively-high ROS levels in this cell line^68^, there were used ZF4 cells previously co-transfected with *crp*-encoding plasmids (CRP-overexpressing cells) in order to potentiate their effect and hence better detect any increase in ROS over baseline levels. Thus, the fluorescence intensity from the ROS reporter probe CM-H2DCFHDA increased in CRP-overexpressing cells (2.9 ± 1.2), in comparison to cells transfected with control plasmid (1.0 ± 0.2, *P* < 0.05) (Fig. 7A). Likewise, by using the Lysotracker Green DND-26 dye it was revealed that the lysosomal pH in CRP-overexpressing cells was more alkaline than in control cells, with fluorescence intensity values of 44.5 ± 1.8 and 49.4 ± 2.1, respectively (*P* < 0.05) (Fig. 7B).

## Discussion

The present work provides evidence on the antiviral activity mediated by CRP1-7, which is mainly due to the induction of a protective state in the host fish cells, rather than to a hampering effect on the viral particles. Evidence showed that the pre-incubation of the host cells with CRP1-7 before the inoculation of the virus is sufficient to inhibit viral infectivity (Figs. 1A and 3A). In this line, the time-independent nature observed in the neutralization properties of most CRP1-7 when co-incubated with the virus also supports this hypothesis and suggests that such antiviral activity is mainly due to the coexistence of CRPs and cells during the adsorption step (Fig. 1B,C); however, an isoform-specific action on viral replication with milder effects cannot be excluded yet. In addition, the inability of CRP1-7 to alter virus binding (Fig. 2A) together its inhibitory effect on viral transcription at 4 h post adsorption (Figs. 2C and 3B) suggests an early blockade of SVCV replication. In this context, only a few cases have been reported in which pentraxins directly interact with the viral particles or viral proteins, such as human SAP^69^ and PTX3^70^ against influenza A virus, but there are numerous studies describing different immunomodulatory properties of pentraxins on different cell types, although never related to antiviral protection^71–73^.

The activation of the IFN system confers an antiviral state to the cells^74^ through the induction of effector molecules capable of limiting viral replication^75^. In this work, evidence showed that CRP1-7 did not trigger the IFN response since the incubation of both EPC and ZF4 cells with CRPs not only did not induce the expression of relevant *mx* or *ifn* isoforms, but they were even repressed in some cases (Figs. 2D and 3C), which is in accordance with other studies in humans^76^. These results were also consistent with the lack of activity observed for the conditioned media from EPC cells treated with CRP1-7 (Fig. 2E).

In contrast, this work demonstrated for the first time that CRPs modulate the autophagic process at several levels, i.e., transcription (Fig. 3D,E), autophagy flux (Fig. 4A) and tissue distribution (Figs. 3E and 4B). Furthermore, this effect was not affected by the presence of SVCV (Fig. 5A,B). In this regard, a recent study using a transgenic approach described significantly reduced autophagy fluxes in the kidney from autophagy reporter mouse lines over-expressing rabbit CRP, and this effect was rescued with rapamycin, which in turn reduced collateral renal injury^77^.

Many viruses, including those of fish, activate/need autophagy to replicate^58,59,66,78–80^. In this regard, there are some previous studies that have analysed the influence of autophagy on SVCV infection^54,57,58^; however, the conclusions of these studies are contradictory with respect to the activation of autophagy as either a negative regulatory mechanism^54,57^ or, as determined more recently, a mechanism required by the virus for replication^58^. In this work, we showed that SVCV requires autophagic activity for replication since the infectivity was neutralized by the autophagy blockers CQ (Figs. 5D) and 3-MA (Fig. 5C,D). In any case, autophagy in fish has been under study only recently, and therefore, there is the possibility of data misinterpretation in pioneering studies.

Additionally, in this work, other autophagy blocking assays were carried out using CQ, and the results support those found with 3-MA. Such inhibition of SVCV replication was potentiated when the autophagy blockers were used in combination with the CRPs, MBCD or 25-HOC. Therefore, together with the decrease in the neutralization of the infection, the results from the combination of each of these three compounds with the autophagy-enhancer rapamycin^55^ indicate that the inhibition of SVCV infection observed when cells were treated with the CRPs, MBCD or 25-HOC is due to the blockade of either autophagy or an element common to the autophagy and viral endocytosis pathways, as has also been reported previously for the rabies virus^56^. Since CRP treatment of the cells resulted in an accumulation of autophagosomes, we suggest that the inhibitory effect on autophagy occurs at a late stage such that it affects the fusion of autophagosomes and lysosomes in a fashion similar to CQ^61,67,81^.

Considering that lysosomes are vulnerable to oxidative stress^82^, to understand the mechanism by which CRPs, 25-HOC and MBCD might block the fusion of the autophagosome/intermediate endosome/amphisome with the lysosome, the possible involvement of ROS in this process was analysed. The results showed a significant reduction in the antiviral effect of each of the three compounds after treatment with the oxidative stress inhibitor NAC (Fig. 6D)^66^. In parallel, it was also demonstrated a direct increase of intracellular ROS levels in CRP-overexpressing ZF4 cells (Fig. 7A), what altogether suggests that the blocking effect on autophagy is mediated by increasing ROS levels. Such a mechanism has been described for other autophagy inhibitors^67^. Briefly, an increase of the ROS concentration induces an increase of the lysosomal pH to inhibit both the fusion of the lysosome with the autophagosome^67^ and the fusion conformation of the SVCV G protein that enables the viral particles to enter the host’s cytosol^49,83^. Indeed, such decrease in lysosomal pH is also observed in CRP-overexpressing ZF4 cells in the present study (Fig. 7B). Furthermore, these results are supported by the aforementioned downregulation of the IFN system observed in response to CRP1-7, since it has also been described that the induction of the antiviral activity of the IFN system is sensitive to the pH of lysosomes/endosomes and, therefore, to CQ treatment^84^.

**Figure 8:**
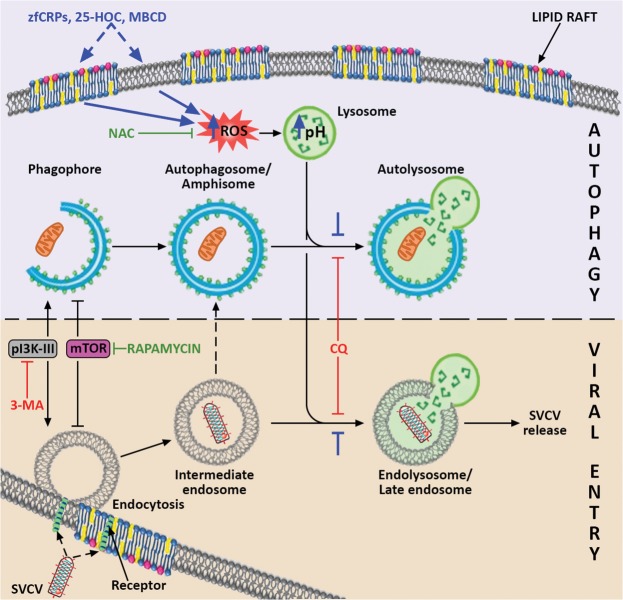
Proposed model for the mechanism by which CRPs, 25-HOC and MBCD interact with autophagy and SVCV entry. It is suggested that these three compounds (their proposed effects are indicated in blue) produce an imbalance in the membrane cholesterol of the lipid rafts, which induces the increase of intracellular ROS. In turn, ROS stimulate the increase in lysosomal pH, which reduces both the fusion of lysosomes and intermediate endosomes (indicated with blue stoppers), and consequently the formation of late endosomes/endolysosomes. Because of their low pH, SVCV requires the formation of endolysosomes to trigger the fusion conformation of the SVCV G protein for viral entry, and a blockade of endolysosomes thus impairs SVCV release into the host’s cytosol. The scheme shows that SVCV endocytic and autophagy pathways share common elements that enable the action of particular autophagy modulators on both of them. The convergence of pathways that may result in the formation of amphisome, as described for other viruses, is also indicated. The positive regulators of both routes are drawn in green, and the negative regulators are presented in red. Artwork drawn and provided by Mr. Diego Sanz.

Therefore, in this work, we propose that CRPs, MBCD and 25-HOC increase the levels of intracellular ROS because of the sequestration/imbalance of membrane cholesterol, which has already been described to induce the formation of ROS^85,86^. For CRPs, this action might be mediated by their multifunctional phosphorylcholine-binding site^87^, which affinity for cholesterol^88^ we recently found to be also conserved in zebrafish CRPs^47^. In fact, the induction of ROS generation as a consequence of the interaction of the monomeric form of human CRP with lipid rafts in human and rat peripheral blood mononuclear cells has also been observed^89^. Thus, the high affinity for cholesterol described for MBCD^64,65^ and CRPs^47,88,90^ suggests that they may have cholesterol-sequestering faction that blocks the ROS-dependent autophagy. Furthermore, 25-HOC added to cells also changes the lipid raft composition with similar inhibition of autophagy. Along this line, the modulation of autophagy in response to an exogenous lipid load has already been demonstrated both *in vitro* and *in vivo*^91^. In fact, the treatment of ZF4 cells with cholesterol significantly increased the amount of intracellular autophagosomes and inhibited SVCV infectivity in a manner similar that of 25-HOC (Supplementary Fig. S5A,B). Moreover, this inhibition was reversed by the use of cholesterol in combination with the cholesterol-sequestering MBCD (Supplementary Fig. S5B). Therefore, we hypothesize that any imbalance in the cholesterol content of the host’s cellular membrane affects ROS generation and consequently disturbs both the autophagic and SVCV replication processes (Fig. 8).

Among the multiple physiological properties of some oxysterols, the ability of 25-HOC^62,92–95^ and 27-HOC^96^ to inhibit viral infections are among the best described. According to our results, 25-HOC, as well as CRP2-6 and MBCD, inhibits the replication of SVCV *in vitro* by a mechanism related to ROS generation and autophagy. Nevertheless, treatment of cells with 25-HOC prior to infection with enveloped viruses blocks the fusion of the viral and cell membranes^62,97^. This fact fits with our proposed model since ROS generation both increases the lysosomal pH and reduces the lysosomal fusion capacity with autophagosomes and endosomes, thus limiting the pH-dependent fusogenic ability of the SVCV G protein.

In summary, this work proposes (Fig. 8) that SVCV requires activation of some of the autophagy machinery to complete its entry steps into the host. Additionally, the treatment with either CRP2-6, 25-HOC, MBCD or any of their combinations is expected to induce the generation of ROS via a change in the cholesterol levels of the host cell membranes that increases lysosomal pH as a consequence. Then, SVCV replication is expected to be reduced not only because of the lowered pH-dependent fusogenic capacity of the SVCV G protein but also because of the reduced rate of fusion of lysosomes with autophagosomes/intermediate endosomes/amphisomes. Since there is evidence of the conservation of these mechanisms in higher vertebrates, this study may be pioneering in the redirection of a research with the potential for a wide range of therapeutic applications.

## Materials and Methods

### Cell lines and virus

EPC cells from the fat-head minnow, the most widely used cell line for research on fish viruses and the diagnosis of fish viral diseases, were purchased from the American Type Culture Collection (ATCC, Manassas, VA, USA, Ref. No. CRL-2872)^51^. The EPC cell monolayers were grown in Dutch-modified Roswell Park Memorial Institute (RPMI) 1640 culture medium (Sigma, St. Louis, USA) supplemented with 10% foetal bovine serum (FBS) (Sigma), 2 mM glutamine, 1 mM sodium pyruvate and 50 μg/mL of gentamicin and 2 μg/mL of fungizone (Gibco BRL-Invitrogen, Carlsbad, CA, USA). The zebrafish embryonic fibroblast ZF4 cell line was purchased from the ATCC (Ref. No. CRL-2050). The ZF4 cells were cultured in Dulbecco’s modified Eagle’s medium (DMEM, Gibco BRL-Invitrogen) supplemented with 10% FBS and 100 µg/mL of Primocin (InvivoGen, San Diego, CA, USA). Both cell lines were maintained at 28 °C in a 5% CO_2_ atmosphere.

The SVCV isolate 56/70 from the common carp was replicated in EPC cells at 22 °C in an atmosphere without CO_2_ in the previously described growth medium but with 2% FBS (infection medium). After 7 days post infection, the supernatants from the infected cells were collected, clarified by centrifugation at 4,000 g and 4 °C for 30 min, aliquoted and stored at −80 °C until use. Virus titres were determined by the focus forming assay as described below.

### Animals

The adult XL wild type zebrafish of 700–900 mg body weight (3–4 cm long) and embryos from transgenic GFP-LC3 zebrafish were obtained by natural spawning from mating adults at one of the host institution facilities (Instituto de Investigaciones Marinas-CSIC, Vigo, Spain). The fish were maintained at 28°C in 30 L re-circulating water tanks by following established protocols^98^. Prior to handling, the fish were anaesthetized by immersion in 100 mg/L tricaine methanesulfonate (MS-222) (Sigma). End-point fish euthanasia was performed by overdose of 500 mg/L.

All experimental procedures with live zebrafish were performed in accordance with the Spanish Law for Animal Experimentation (Royal Executive Order, 53/2013) and the European Union directive 2010/63/UE. Animal experimental procedures were approved by the local government ethics committee on animal experimentation (Dirección General de Agricultura, Ganadería y Pesca, Generalitat Valenciana), and the Project Evaluation Board of Miguel Hernández University (permit no. UMH.IBM.JFG.01.14), as well as the CSIC National Committee on Bioethics under approval number ES360570202001/16/FUN01/PAT.05/tipoE/BNG.

### Production of enriched, depleted and conditioned CRP1-7 supernatants

The pMCV1.4 plasmids encoding each *crp1-7* from our previous studies^42,47^ were used as described to obtain cell-free supernatants enriched in the CRP1-7 isoforms from EPC cells 4 days after transfection. Likewise, the CRP content was characterized by ELISA, western blotting and cholesterol-binding affinity^42,47^. Similarly, the pMCV1.4 constructs with genes encoding either *gfp* or zebrafish *il6* were used to obtain control supernatants without CRPs and supernatant enriched in IL-6. For some experiments, a solution of equally mixed CRP2-6 supernatants (CRP-mix) was used. All supernatants were stored at −80 °C until use.

To demonstrate that the antiviral activity of the CRP1-7 supernatants was due to the CRP1-7 proteins rather than to other possible CRP-induced EPC-derived compounds, the supernatants were CRP depleted by incubating them with solid-phase immobilized 25-HOC (Sigma), a lipid for which most CRP1-7 showed the highest affinity in our previous work^47^. Briefly, the wells in Maxisorb 96-well plates (Nunc, Roskilde, Denmark) were coated to dryness with ethanol-dissolved 100 µM 25-HOC and were kept dried until use. Then, after washing them 3 times with phosphate buffered saline (PBS), 100 µL of the 4-fold-diluted CRP1-7 supernatants were added per well and incubated for 2 h. Finally, the depleted supernatants were collected and stored at −80 °C until use.

To produce the CRP-conditioned supernatants, the EPC cell monolayers were incubated for 2 h at 22 °C with CRP1-7; after 3 washes with EPC growth medium, fresh EPC growth medium was added, and the cells were incubated for another 24 h at 22 °C. Finally, these supernatants were collected, clarified as described, aliquoted and stored at −80 °C until use.

### SVCV infection *in vitro* assays

To explore the effects of the experimental treatments on the replication of SVCV, several different infection assays were performed on the EPC and ZF4 cell. In general, the cells grown on the 96-well plates were inoculated with SVCV supernatants in infection medium at an MOI of 10^−2^ SVCV per cell (unless stated otherwise) and incubated together for 2 h at a temperature of 4 °C (the low temperature was chosen to delay viral replication during the initial adsorption/binding step and synchronize viral replication). Then, the viral inoculants were removed, and the EPC cell monolayers were washed 3 times with infection medium to eliminate the unattached SVCV particles. Subsequently, fresh infection medium was added, and plates were further incubated for 20 h at 22 °C.

Variations in this common procedure were used to investigate the potential interactions of CRPs with either the EPC cells or the SVCV. Thus, such variations were made by incubating (i) CRP1-7 with SVCV or EPC cells before viral adsorption (pre-adsorption treatments) and (ii) CRP1-7 and cells together during the SVCV adsorption step (adsorption treatment) and (iii) by adding CRP1-7 after the SVCV adsorption step (post-adsorption treatment). Diagrams describing such experimental designs are shown in Fig. 1A. After every incubation step, the cell monolayers were washed 3 times with infection medium.

### SVCV focus forming assay

To assess the effect of the treatments on viral infectivity *in vitro*, SVCV-infection foci of 5–20 cells were immune-labelled to be quantified as previously described^99^. Briefly, at 20 h post adsorption, the cell monolayers were fixed with 4% formalin (Sigma) in PBS for 20 min and then incubated for 24 h at 4 °C at a 1:300 dilution with polyclonal anti-SVCV (BioX Diagnostics SA, Jemelle, Belgium) in antibody (Ab)-dilution buffer made of PBS containing 1% bovine serum albumin (BSA), 1% goat serum and 0.5% Triton X-100 (Sigma). After 3 washes with PBS, there was another incubation period with a FITC-labelled goat anti-mouse antibody (Sigma) diluted 1:300 in Ab-dilution buffer for 45 min at room temperature and protected from light. Finally, the cell monolayers were washed 3 times with PBS again, and immunofluorescence-labelled foci were counted or photographed by means of a fluorescence DMI 3000B inverted microscope with an EL6000 compact light source and a DFC3000G digital camera (Leica, Bensheim, Germany). The data are expressed as percentages of neutralization based on calculations with the formula: 100 - (number of foci in the treatment samples/number of foci in the control samples) × 100.

### G protein-mediated fusion assays in SVCV-infected EPC cell monolayers

To assess the effect of the treatments on the ability of the SVCV surface G protein to fuse membranes, the G protein-dependent fusion activity was induced by lowering the pH of the infected EPC cell monolayers and quantified by counting syncytia as previously described^99^. Briefly, at 20 h post adsorption, the medium was removed from the SVCV-infected EPC cell monolayers, which were washed 3 times with infection medium and then treated with CRP1-7 for 2 h at 22 °C. After another 3 washes, G protein-dependent fusion was triggered by incubating the EPC cell monolayers with infection medium at pH 6 (fusion medium) for 30 min, washing them again 3 times and subsequently incubating them with infection medium at pH 7.5 for 2 h at 22 °C. Finally, the cell monolayers were fixed by the application of cold methanol (−20 °C) for 15 min, air dried, stained with Giemsa (5 mg/mL in PBS), washed 3 times with PBS and air dried. The syncytia resulting from the fusion of adjacent cells were then counted and photographed with the aforementioned microscope. The percentage of syncytia production from G protein mediation was calculated by the following formula: 100 × number of syncytia in treated EPC cell monolayers/number of syncytia in control (GFP-treated) EPC cell monolayers. Three different assays, each in triplicate, were performed per experiment. The results are shown as the mean and standard deviation (s.d.).

### Assay of SVCV binding to the EPC cell monolayers

To study whether CRP1-7 inhibited the binding of SVCV to the EPC cells, the SVCV supernatants (MOI of 1) in the presence of CRP1-7 or GFP were incubated with the EPC cell monolayers during the adsorption step (2 h at 4 °C) and then washed 3 times with infection medium to remove the unattached SVCV. Thereafter, the extent of the cell-bound SVCV was estimated by measuring the number of viral genomes derived from the detection copies of the SVCV *n* gene (primer sequences are shown in Supplementary Table S1) by RT-qPCR as described later.

### Determination of SVCV replication levels in EPC cells at early stages post adsorption

To determine whether CRPs affect SVCV replication at early stages post adsorption, both EPC and ZF4 cell monolayers were incubated with the CRP-mix for 2 h at 22 °C. Then, the cells were washed 3 times with infection medium and inoculated with SVCV at an MOI of 10^−2^ for an additional 2-h incubation at 4 °C. After another 3 washes, fresh infection medium was added, and the plates were further incubated at 22 °C. The infected cells were collected at 0, 1, 2, 3, 4 and 5 h post adsorption for the subsequent analysis of their viral replication levels by performing RT-qPCR on the SVCV *n* and *g* gene transcripts (primer sequences are shown in Supplementary Table S1).

### Analysis of the transcriptional modulation of the interferon (IFN) system and autophagy

To assess whether CRPs affected the IFN system and/or autophagy at the transcription level, EPC cells were treated with CRP1-7 for 2 h at 22 °C, washed 3 times with infection medium and further incubated at 22 °C. The samples were collected at 20 h post treatment for the subsequent RT-qPCR analysis of the transcripts of *mx*, an IFN-stimulated gene commonly used as one of the best reporters of an IFN system response^50^. A similar procedure was followed with ZF4 cells except the CRP-mix was used, and the samples were collected at 1, 2, 3, 4, 5 and 20 h post treatment. The genes analysed in the latter case were the *mx* paralogs *mxa* and *mxe*, the IFNφ coding genes *ifnphi1* and *ifnphi2*, and the autophagy-related *beclin1*, *lc3a*, *wipi1*, *atg5*, *gabarap* and *ambra1* genes (primer sequences are shown in Supplementary Table S1).

### Injection of the CRP-mix and IL-6 into adult zebrafish

Four adult zebrafish were i.p. injected with 5 µL of GFP, CRP-mix or IL-6 supernatant. Two days post injection, the spleen, liver and head kidney tissues were individually dissected, immersed in RNA later (Ambion, Austin, TX, USA) and stored at - 80 °C until they were later analysed by RT-qPCR (primer sequences are shown in Supplementary Table S1) as described below.

### RNA isolation, cDNA synthesis and qPCR

Total RNA was extracted from cultured cells and organ tissue using the E.Z.N.A. HP Total RNA and E.Z.N.A. HP Tissue RNA kits (Omega Bio-tek, Norcross, GA, USA), respectively. The samples were then treated with DNase (Turbo DNA‐free™ Kit, Ambion Inc., Austin, TX, USA), to eliminate residual genomic DNA, by following the manufacturer’s instructions. Each cultured cell sample was obtained by pooling four of the 96-wells in the plates. RNA concentrations were estimated with a Nanodrop 1000 spectrophotometer (Thermo-Fisher Scientific, Waltham, MA, USA). Isolated RNA samples were stored at −80 °C until use.

For the synthesis of cDNA, 0.5 μg of isolated RNA from each sample was used. Moloney murine leukaemia virus (M-MuLV) reverse transcriptase (Gibco BRL-Invitrogen) was used as previously described^36^.

qPCR was performed by using an ABI PRISM 7300 thermocycler (Applied Biosystems, NJ, USA). The reactions were conducted in 20 μL of reactants, including 2 μL of cDNA, 900 nM forward and reverse primer corresponding to the cDNA (Sigma) (primer sequences are shown in Supplementary Table S1) and 10 μL of SYBR Green PCR master mix (Life Technologies, Paisley, UK). Non-template controls were added for each gene analysis. All reactions were performed using technical duplicates. The cycling conditions included an initial denaturing step (10 min at 95 °C), followed by 40 cycles for 1 min at 65 °C and for 1 min at 95 °C, and an extension step of 10 min at 65 °C. The melting curves for each reaction were checked for inconstancies. The results were obtained by normalizing the number transcripts of each target gene to the corresponding endogenous reference transcripts (transcripts of the *ef1a* gene for the EPC cells and 18S ribosomal RNA for the zebrafish tissues) from the same sample. A variation of Livak and Schmittgen’s method^100^ with the formula 2^Ct ref. – Ct target^ was used. The results were normalized to the expression of the corresponding housekeeping gene transcription and, when stated, relative to the control samples calculated by the following formula: transcript levels in treated samples/transcript levels in control samples.

### Immunofluorescence assays and confocal microscopy

For these experiments, several compounds were selected because of either their anti-SVCV activity (25-HOC (C_27_H_46_O_2_)), their interaction with membrane cholesterol to affect balance (MBCD, 25-HOC and cholesterol (C_27_H_46_O)) or their autophagy-modulating properties (CQ, 3-MA, NAC and rapamycin), all of which were provided by Sigma. Stock solutions (40 mM MBCD in PBS; 0.4 mg/mL of 25-HOC and cholesterol in ethanol; 0.1 M CQ in H_2_O; 0.6 M NAC in H_2_O and 0.2 M 3-MA in H_2_O) were stored at -20°C until use.

The ZF4 monolayers grown to 80% confluence on 24-well plates with 12-mm glass coverslips were treated with the following compounds in 500 μL of ZF4 infection medium for 4 h at 22°C: CQ (25 µM), 3-MA (10 μM), 10 μg/mL of 25-HOC (including 2.5% ethanol), MBCD (4 mM), 10 μg/mL of cholesterol (including 2.5% ethanol), SVCV (MOI of 1), GFP and CRP-mix, and the combinations CRP-mix (or GFP) with either SVCV, 25-HOC or MBCD. Non-treated cells were also included as a control. After treatment, the cells were washed 3 times with infection medium and fixed with 2% formalin for 15 min at 4°C. After 3 washes with PBS, the cells were blocked with 1% BSA and 0.5% Triton X-100 (Sigma) (blocking buffer) in PBS for 1 h, washed again and then incubated overnight at 4 °C at a 1:200 dilution in a blocking buffer with mouse anti-LC3B monoclonal antibody (NanoTools Antikörper technik GmbH & Co., Teningen, Germany). After washing, the cells were incubated with the secondary antibody Alexa Fluor^®^488 goat anti-mouse IgG (1:500 dilution in blocking buffer) for 1 h at room temperature and stained with 0.1 µg/mL of the DNA-specific dye 4,6-diamidino-2-phenylindole (DAPI) solution (Molecular Probes-Life Technologies, Paisley, UK) for nuclear localization. Finally, cell samples were washed 3 times and mounted using ProLong Antifade Reagents (Life Technologies). Confocal images were captured by using a TSC SPE confocal microscope and LAS AF software (all Leica).

### Determination of intracellular autophagosomes

The quantification of autophagosomes was carried by analysis of the immunofluorescence images with ImageJ v1.52a software (US National Institutes of Health, Bethesda, MD, USA). For this determination, the FITC-induced fluorescence from each image was measured by applying a threshold of 25 brightness in the green spectra, which excluded the background but selected the fluorescence-labelled puncta. DAPI-stained nuclei were counted manually. The data are presented, after normalization, as the selected fluorescent area per cell for each treatment compared to the control by the following formula: average fluorescent area for each cell from treated monolayers/average fluorescent area for each cell from control (GFP-treated) monolayers. For each treatment, three images were analysed from two different experiments (approximately 100 cells were analysed per treatment).

### Visualization of GFP-LC3-recombinant zebrafish embryos previously injected with crps and il6 transgenes

To test the effects of CRPs and IL-6 in the process of autophagy *in vivo*, groups of 30 one-cell-stage embryos of GFP-LC3 zebrafish^101^ were microinjected with 2 nL of PBS containing 150 pg of either pMCV1.4, pMCV1.4-*crp1, 4 or 5* or pMCV1.4*-il6*. The microinjections were performed with glass microcapillary pipettes (WPI, Sarasota, FL, USA) incorporated into an MN-151 micromanipulator and an IM-30 microinjector (Narishige, Tokyo, Japan). The treated 3-day-old hatched larvae were anaesthetized (by adding 200 µL of 0.05% MS-222 solution to a Petri plate with 10 mL of water) and photographed using a Multi‐Zoom AZ100 microscope equipped with a DS-Ri1 digital camera (Nikon, Melville, NY); the images were processed with LAS AF software (Leica).

### Effect of autophagy inhibitors and cholesterol-interacting compounds on SVCV replication

The anti-SVCV activity of the CRPs, 25-HOC and MBCD was compared in the presence and absence of some relevant autophagy modulators (in particular, 3-MA, CQ, rapamycin and NAC). Briefly, EPC monolayers at 22 °C were first incubated with either 3-MA (1 mM and a 0–1 mM gradient, 20 h), CQ (25 μM, 30 min), rapamycin (25 μM, 4 h) or NAC (1 mM, 20 h), washed 3 times with infection medium and then treated for 2 h with either GFP, CRP-mix, 10 μg/mL of 25-HOC or 1 mM MBCD. Similarly, the effect of cholesterol on SVCV infectivity was assessed with and without MBCD. For this analysis, the EPC cell monolayers were treated with MBCD (1 mM), cholesterol (0.5 and 1 mM) or MBCD (1 mM) with cholesterol (either 0.5 or 1 mM) for 2 h at 22 °C. The treated EPC cell monolayers were then washed with infection medium 3 times and infected with SVCV (MOI of 10^−2^) for the subsequent determination of the number of foci of infection as described above.

### Oxidative stress detection

In order to verify if the exposure of ZF4 cells to CRPs leads to the generation of intracellular ROS, there was used a chloromethyl derivative of 2′,7′-dichlorodihydrofluorescein diacetate (CM-H2DCFDA, Molecular Probes, Leiden, The Netherlands) as general oxidative stress indicator. CM-H2DCFDA passively diffuses into cells where is deacetylated by intracellular esterases producing the non-fluorescent DCF product. DCF is oxidized by ROS in the fluorescent H2DCFDA, which can be detected using fluorescence microscopy at 485 nm (excitation) and 535 nm (emission) wavelengths. Briefly, ZF4 cells (10^6^) were transfected with 7 μg of each expression plasmid encoding zebrafish *crp* (pMCV1.4-*crp1-7*), the empty plasmid (pMCV1.4) or co-transfected with 1 μg of each *crp*-encoding plasmid (CRP-mix) using the Neon Transfection System (Invitrogen, Carlsbad, CA, USA) (settings: 1400 V, 20 ms, one electric pulse). After transfection, cells were resuspended in 0.5 mL of DMEM containing 10% FBS and 100 μL/well were seeded into 96-well black opaque plates. After 48 h of incubation at 28°C, cell culture medium was removed, monolayers were washed with PBS and incubated with 5 μM CM-H2DCFDA for 30 min at 28 °C in dark conditions. The fluorescence for each experimental condition was measured using a plate reader fluorometer (Fluoroskan Ascent FL, Labsystems, Helsinki, Finland). CM-H2DCFDA was also added to empty wells as a background control. The background mean value was subtracted to each fluorescence measure obtained. There were performed 4 independent replicates for this experiment.

### Evaluation of intracellular pH changes

In order to evaluate if CRPs can change the pH of the lysosomes in the ZF4 cell line, cells were incubated with the cell permeable green dye LysoTracker Green DND-26 (Cell Signaling Technology, Danvers, MA, USA), which stains acidic compartments (lysosomes) in live cells. Briefly, ZF4 cells were co-transfected with each *crp*-encoding plasmid (CRP-mix) or with just the empty plasmid (pMCV1.4) using the same methodology described above but seeding the cells into 24-well plates. After 48 h post-transfection, monolayers were incubated with 60 nM Lysotracker Green DND-26 for 30 min at 28 °C in dark conditions. Thereafter, monolayers were washed with PBS and then trypsin-detached. The fluorescence of each condition was measured by flow cytometry (FACSCalibur flow cytometer, BD Biosciences, San Jose, CA, USA). Each treatment was evaluated in 6 different samples.

### Statistical analysis

The data are shown as the mean and s.d. The resulting data sets were subjected to the most appropriate statistical analysis depending on each particular experimental design. The differences between two groups from the same data set were analysed by two-tailed unpaired Student’s t-test or multiple Student’s t-tests corrected by using the Holm-Sidak method, and one- and two-way ANOVA and Sidak’s multiple comparison tests were used to determine differences between groups. Prism v7 (GraphPad software, La Jolla, CA) was used for creating the graphs and performing statistical analysis. The significant differences, as determined by *P* < 0.05, *P* < 0.01 and *P* < 0.001, are indicated as a, b and c, respectively, when the data are compared to those of the corresponding control groups.

### Graphics

The image processing and diagram drawing were undertaken with Adobe Photoshop CC 2017 (Adobe Systems Inc., San Jose, CA, USA).

## Supplementary information

Supplementary information.
