# Supplementary material for: Zebrafish C-reactive protein isoforms inhibit SVCV replication by blocking autophagy through interactions with cell membrane cholesterol
Source: Sci Rep. 2020 Jan 17;10:566. doi: 10.1038/s41598-020-57501-0 (PMC6969114; doi:10.1038/s41598-020-57501-0)
Supplement: Supplementary file 1 — Supplementary information. [file 41598_2020_57501_MOESM1_ESM.pdf]

Supplementary Information

Zebrafish C-reactive protein isoforms inhibit SVCV replication by blocking autophagy through interactions with cell membrane cholesterol

Melissa Bello-Perez, Patricia Pereiro, Julio Coll, Beatriz Novoa, Luis Perez and Alberto Falco

[1] Instituto de Investigación, Desarrollo e Innovación en Biotecnología Sanitaria de Elche (IDiBE), Miguel Hernández University (UMH), Elche, 03202, Spain.

[2] Instituto de Investigaciones Marinas (IIM), Consejo Superior de Investigaciones Científicas (CSIC), Vigo, 36208, Spain.

[3] Instituto Nacional de Investigaciones y Tecnologías Agrarias y Alimentarias (INIA), Dpto. Biotecnología, Madrid, 28040, Spain.

Table S1. Primer sequences used for qPCR.

Organism	Gene	Sequence (5' - 3')	Accession. no.
P. promelas	***ef1a***	Fw: CTGGAGGCCAGCTCAAACAT<br>Rv: CATTTCCCTCCTTACGCTCAAC	AY643400
	mx	Fw: GGAGAAGAGGTTAAATGTGGATCAG<br>Rv: TGAAGTGCCTTTTTATCTTAATCT	KM099175
D. rerio	**18S**	Fw: ACCACCCACAGAATCGAGAAA<br>Rv: GCCTGCGGCTTAATTTGACT	NR_145818
	ef1α	Fw: CCACGTCGACTCCGGAAA<br>Rv: CGATTCCACCGCATTTGTAGA	AY422992.1
	ambra1	Fw: TCTTTCGAGAAATGGCACCT<br>RV: CTCTCTGCGTTAGGGACAGG	XM_002667669
	atg5	Fw: AGAGAGGCAGAACCCTACTATC<br>Rv: CCTCGTGTTCAAACCACATTTC	NM_205618
	beclin1	Fw: GATCATGCGCAATGGTGGCTTTC<br>Rv: CCTCCTGTGTCCTCAATCTTT	AB266448
	gabarap	Fw: GTCTGACCTCACAGTTGGGC<br>Rv: TCCTGGTAGAGCAGTCCCAT	NM_001013260
	lc3a	Fw: CAATCAGCACAGCATGG<br>Rv: GTAAAGGAAGCCGTCTTC	NM_214739
	wipi1	Fw: GTGAGAGGGTAGAGAACAG<br>Rv: GTAACAACGACCCAACATC	XM_005164002
	mxa	Fw: CACAGACAATCATGCCACCT<br>Rv: TTTGCAGCTCCAAAGCAGCT	NM_182942
	mxe	Fw: AGTCACCCAATGTCAGTGCA<br>Rv: GCTGAGAGATGTACTGGTTC	BC095587
	ifnphi1	Fw: GAGCACATGAACTCGGTGAA<br>Rv: TGCGTATCTTGCCACACATT	BC162493.1
	ifnphi2	Fw: CCTCTTTGCCAACGACAGTT<br>Rv: CGGTTCCTTGAGCTCTCATC	BX005440.4
**SVCV**	***n-SVCV***	Fw: GCATTATGCCGCTCCAAGAG<br>Rv: AGCTTGCATTTGAGATCGA	U18101
	g-SVCV	Fw: TACAGATTCGGGGGATCTTG<br>Rv: ACCAACGTTCCATCAACACA	NC_002803

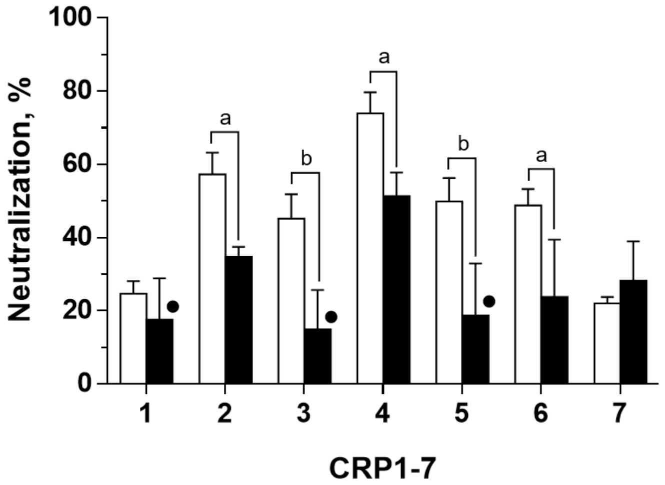

Supplementary Figure S1. Antiviral activity of the CRP1-7-depleted supernatants. Each CRP1-7-containing supernatant was CRP depleted by incubation with immobilized 25-HOC on ELISA plates for 2 h. Whole CRP1-7 (white bars) and depleted CRP1-7 (black bars) were incubated with EPC cells for 2 h prior to SVCV adsorption (MOI of 10^{-2}). SVCV infection was determined by the focus forming assay. The data are expressed as percentages of neutralization by the following formula: 100 - (number of fluorescent foci in the treatment group / number of fluorescent foci in the control GFP group) × 100. The data are presented as the mean and s.d. of the two independent experiments, each performed in triplicate. • indicates no significant differences between the treatment and the GFP control. Statistically significant differences are indicated as in Fig. 1. Data were analysed by using two-way ANOVA with Sidak's multiple comparisons test.

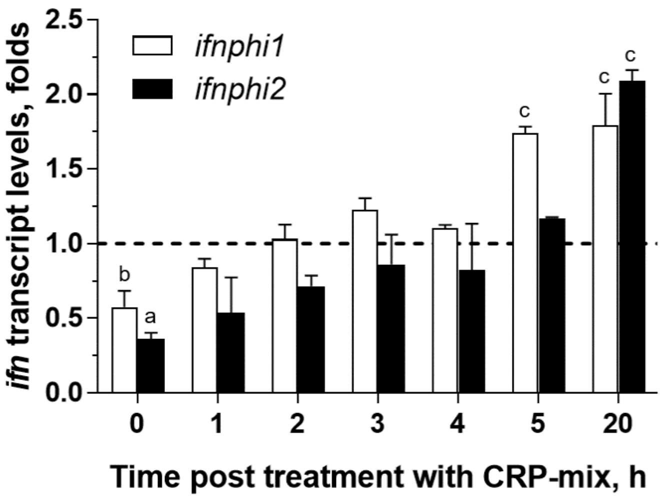

Supplementary Figure S2. Assessment of the ability of CRP-mix to induce the transcription of *ifnphi1* and *ifnphi2 in vitro*. The transcript levels of *ifnphi1* and *ifnphi2* in the ZF4 cells treated with CRP-mix for 2 h prior to SVCV inoculation at different times post adsorption were quantified by RT-qPCR. The data are expressed as fold changes relative to GFP treatments by the following formula: *ifn* expression in CRP-mix-treated monolayers / *ifn* expression in GFP-treated monolayers. This experiment was performed twice in quadruplicate, and the results are presented as the mean and s.d. The statistically significant differences in comparison to the GFP-treatment group are as indicated in Fig. 1. Data were analysed by using two-way ANOVA with Sidak's multiple comparisons test.

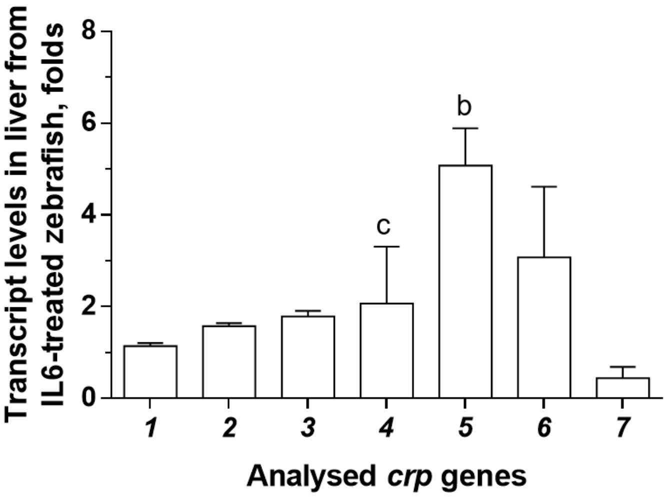

Supplementary Figure S3. Assessment of the ability of IL-6 to induce the expression of *crp1-7* transcripts *in vivo*. Four adult zebrafish were i.p. injected with supernatants enriched in IL-6. Two days post injection, the transcript levels of each *crp1-7* isoform from the liver were quantified by RT-qPCR and normalized to 18S ribosomal levels from the corresponding samples. The data are expressed as fold changes relative to GFP treatments by the following formula: *crp1-7* expression in the liver of zebrafish injected with IL-6 / *crp1-7* expression in the liver of zebrafish injected with GFP. The results are represented as the mean and s.d., and the statistically significant differences in comparison to the GFP-treated group are as indicated in Fig. 1. Data were analysed by multiple Student's t-tests by the Holm-Sidak method.

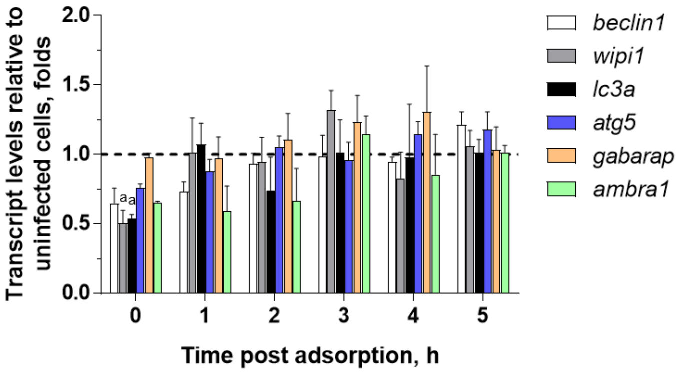

Supplementary Figure S4. Transcriptional modulation of autophagy by SVCV in the ZF4 cells. The transcript levels of the genes of relevant autophagy elements (*beclin1*, *wipi1*, *lc3a*, *atg5*, *gabarap* and *ambra1*) at different times post adsorption (0-5 h) in ZF4 cells treated with GFP for 2 h prior to viral inoculation (MOI of 1) were quantified by RT-qPCR. This experiment was performed twice in quadruplicate. The data are expressed as indicated in Fig. 3 (relative to the uninfected group). The statistically significant differences in comparison to the control group are as indicated in Fig. 1. Data were analysed by two-way ANOVA with Sidak's multiple comparisons test.

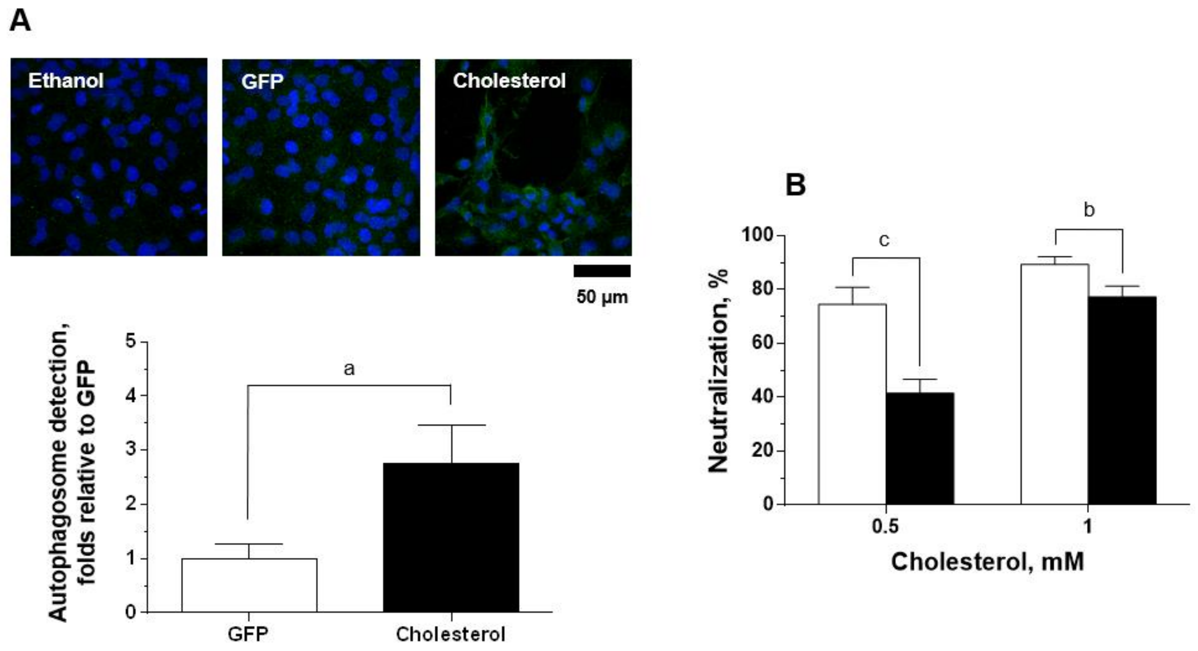

Supplementary Figure S5. Effect of the addition of exogenous cholesterol on intracellular autophagosome levels and SVCV infection *in vitro*. (**A**) Representative confocal images of FITC antibody-labelled LC3B in ZF4 cells treated with GFP and cholesterol, as well as ethanol (solvent control of both 25-HOC and cholesterol), for 4 h. Nuclei were stained with DAPI. Autophagosome levels were quantified as described in Fig. 4 and in the methods. The scale bar is equal to 50 µm. (**B**) Effect of cholesterol in the absence and presence of MBCD on SVCV replication *in vitro*. SVCV infectivity was assessed for EPC cells treated with cholesterol (0.5 and 1 mM) together with MBCD at either 0.5 mM (white bars) or 1 mM (black bars) for 2 h before infection. SVCV infection was determined by the focus forming assay. The results are represented as percentages of neutralization relative to the untreated group. This experiment was performed 3 times in triplicate. Statistically significant differences between cholesterol treatments with and without MBCD are indicated as in Fig. 1. Data were analysed by using two-tailed unpaired Student's t-test.
